# Dramatic response of advanced pulmonary sarcomatoid carcinoma to tislelizumab combined with anlotinib: a case report

**DOI:** 10.3389/fonc.2025.1531700

**Published:** 2025-02-07

**Authors:** Ranran Li, Xiliang Di, Yuan Li, Hao Li, Chonghua Liu

**Affiliations:** ^1^ Department of Oncology, The Linyi County People’s Hospital, Dezhou, Shandong, China; ^2^ Department of Emergency, The Linyi County People’s Hospital, Dezhou, Shandong, China

**Keywords:** pulmonary sarcomatoid carcinoma, tislelizumab, anlotinib, case report, immunotherapy

## Abstract

Pulmonary sarcomatoid carcinoma (PSC) is a scarce pathologic type of lung cancer of non-small cell lung cancer (NSCLC) that exhibits resistance to conventional chemotherapy and radiotherapy, resulting in a poor prognosis. Herein, a 67-year-old man was admitted to the hospital in January 2022 with a diagnosis of PSC for over 6 months and a newly discovered brain metastasis for 4 days. He had previously undergone two unsuccessful chemotherapy regimens: bevacizumab combined with pemetrexed and loplatin, and albumin-bound paclitaxel combined with loplatin. Radiotherapy was performed for the brain and skull metastases, followed by treatment with tislelizumab combined with anlotinib, a programmed cell death protein 1 (PD-1) inhibitor with anti angiogenic drug, respectfully. The patient initially received nine cycles of treatment with tislelizumab and anlotinib, resulting in a significant shrinkage of the lung tumor. Subsequently, anlotinib was discontinued due to bleeding in the brain metastasis, and the patient received two additional cycles of tislelizumab. Following improvement in the hemorrhage from brain metastasis, the patient received two cycles of treatment with tislelizumab and anlotinib. Treatment was subsequently interrupted for 1 month due to the coronavirus disease 2019 (COVID-19) pandemic, which then resumed with two additional cycles of tislelizumab and anlotinib. Finally, the patient refused to continue treatment due to the progression of the brain metastasis and economic conditions, despite the stable condition. At this point, the patient has achieved over a year of progression-free survival, with overall survival exceeding 39 months. This case illustrates the efficacy and safety of combining antitumor immunotherapy with anlotinib, a targeted anti-angiogenic therapy.

## Introduction

Pulmonary sarcomatoid carcinoma (PSC) is an uncommon type of malignant lung cancer with a bad prognosis, constituting approximately 0.1%–0.4% of all lung cancer cases (1). This tumor exhibits histological features of both cancer and sarcoma and is characterized by rapid disease progression, high malignancy, and a poor prognosis (2). PSC mainly involves five pathological subtypes, namely, pleomorphic carcinoma (PC), spindle cell carcinoma (SCC), giant cell carcinoma (GCC), carcinosarcoma (CS), and pulmonary blastoma (PB) (3).

At present, there is no specific clinical treatment guideline for PSC, mainly referring to the treatment principles of non-small cell lung cancer (NSCLC). Its insensitivity to traditional radiotherapy and chemotherapy urgently necessitates novel therapeutic targets or methods (4, 5). Immune checkpoint inhibitors (ICIs) have become a first-line treatment option in advanced NSCLC, mainly targeting patients with a high expression of programmed death-ligand 1 (PD-L1), a high tumor mutation burden (TMB), and no effective gene mutations (6). Recent studies have shown that PSC exhibits a high expression level of PD-L1 (7) and an elevated TMB (8), suggesting that patients with PSC might benefit from treatment with ICIs. In addition, patients with PSC typically exhibit significant vascular invasion (blood vessel invasion, BVI) (9), indicating potential benefits from anti-angiogenic therapy. These provide new directions for the combination of immunotherapy and anti-angiogenic therapy in patients with PSC.

This article presents evidence of the significant efficacy of the combination of anlotinib and tislelizumab in treating patients with advanced PSC who have failed two lines of chemotherapy.

## Chief complaints

A 67-year-old man was admitted to our hospital on January 9, 2022, with a diagnosis of lung cancer for over 6 months and brain metastasis for 4 days.

## History of present illness

The patient developed swelling in the right neck for 3 days without obvious cause and came to the Outpatient Department of Linyi County People’s Hospital on July 11, 2021. A neck plain CT scan showed a thickening and low-density lesion in the right common jugular vein, suggesting the possibility of a cancer thrombus. Multiple enlarged lymph nodes on both sides of the neck were considered metastases. A chest plain CT scan indicated a mass-like soft tissue density lesion in the upper right mediastinum, with a maximum cross-section of approximately 7.7 × 7.4 cm, clear boundaries, uneven density, and multiple enlarged lymph nodes in the mediastinum. Imaging diagnosis revealed 1) tumor in the upper right mediastinum and 2) multiple enlarged lymph nodes in the mediastinum. The patient sought further treatment at the Third Hospital of Shandong Provincial Hospital. CT-guided percutaneous lung biopsy was performed on July 12, 2021. Pathological diagnosis (lung biopsy) suggested a poorly differentiated carcinoma, with the immunohistochemical findings consistent with PC, including elements of a poorly differentiated adenocarcinoma and SCC. Immunohistochemistry indicated CD56 (−), CGA (−), CK7 (+), napsin A (lesion+), P40 (−), Syn (−), TTF-1 (lesion+), CK (+), vimentin (mostly+), and Ki-67 (40%–50%+) (**Supplementary Figure S1**). Ultimately, the comprehensive diagnosis was PSC (cT4N2M1, stage IVB).

The patient refused to undergo lung cancer-related gene and PD-L1 testing, as well as other examinations. Consequently, treatment with “bevacizumab 500 mg + pemetrexed 0.8g + loplatin 50 mg” was given for three cycles, with tumor evaluation progressing in early October 2021. Subsequently, three cycles of chemotherapy were administered with “albumin paclitaxel 400 mg + loplatin 50 mg.” The tumor was evaluated as partial remission by chest and abdominal CT in November 25, 2021. The last treatment was on November 25, 2021. On December 5, 2021, a chest and abdominal CT scan showed progression of a mediastinal mass, and no further treatment was administered. Due to the occurrence of weakness in the right limb for 7 days, a complete cranial CT scan was performed on January 5, 2022, which showed metastases in the left frontal parietal junction area and bone destruction in the left parietal bone. On January 6, 2022, cranial MRI revealed a metastatic tumor in the left frontoparietal junction, accompanied by surrounding brain tissue edema and a localized abnormal density lesion in the left parietal bone, suggestive of a metastatic tumor.

## History of past illness

The patient was previously in good physical health.

## Personal and family history

The patient had no history of smoking or drinking and had no family medical history.

## Physical examination upon admission

Physical examination revealed the following: temperature, 36.6°C; pulse, 78 bpm; respiration, 19 breaths/min; blood pressure, 106/76 mmHg; height, 170 cm; weight, 65 kg; body surface area 1.75 square meters; and Karnofsky Performance Status (KPS), 80 points. Examination revealed no palpable superficial lymph node enlargement, diminished respiratory sounds in the right lung, and clear sounds in the left lung, with no significant dry or wet rales bilaterally. The right limb muscle strength was grade IV, and the limb muscle tone was normal. No obvious positive signs were noted in the remaining physical examinations.

## Laboratory examinations

Upon admission on January 9, 2021, no significant abnormalities in the blood routine, liver and kidney function, ion count, and blood coagulation were observed.

## Imaging examinations

To avoid excessive word count restrictions, please refer to “History of present illness” and “Treatment.”

## Diagnosis

Based on the patient’s medical history, symptoms, physical signs, and auxiliary examinations (including pathology and imaging), the diagnosis was right upper mediastinal PSC with metastasis to the left frontal–parietal junction and left parietal bone (cT4N2M1, stage IVB).

## Treatment

**Figure 1:**
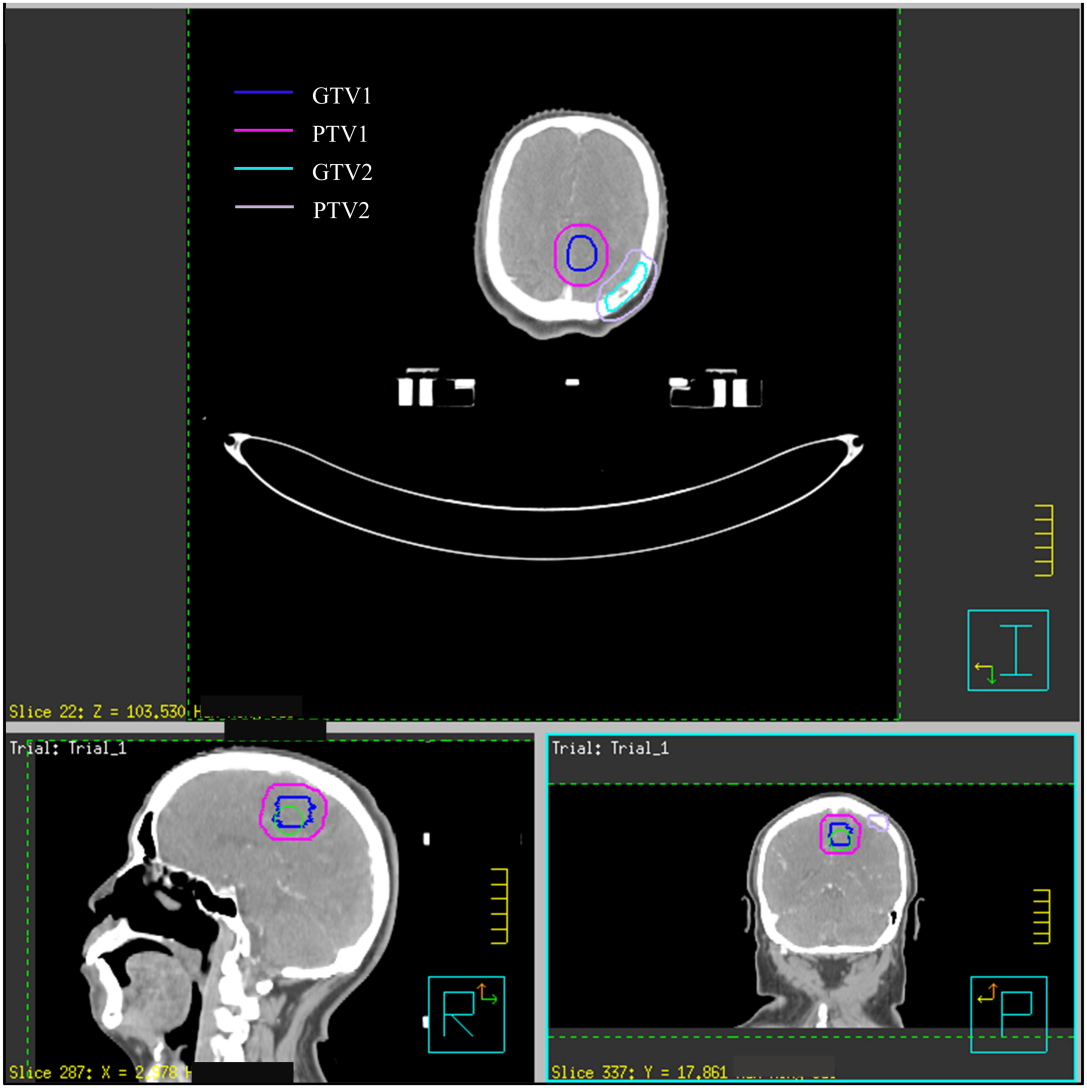
Design of radiotherapy target area.

Upon admission on January 9, 2022, dehydration and intracranial pressure reduction treatments such as mannitol and dexamethasone were given, and the weakness of the right limb improved. Enhanced brain CT location was arranged: A 1.8-cm × 1.6-cm circular lesion was observed in the gray white matter junction area of the left parietal lobe, and an irregular destruction was found in the left parietal bone. Imaging diagnosis revealed brain metastasis and left parietal bone metastasis. Radiotherapy localization and intensity-modulated radiotherapy were further arranged. Specifically, the brain metastatic tumor was characterized as GTV1 (gross tumor volume). GTV1 was uniformly expanded 8 mm as PTV1 (planning target volume), and the prescribed dose was 3.0 Gy × 15 fractions. The skull metastases were characterized as GTV2, with GTV2 defined by a uniform shell of 5 m to PTV2 and a prescribed dose of 3.0 Gy × 10 fractions (**Figure 1**). Radiotherapy commenced on January 12, 2022, and concluded on February 4, 2022, with no obvious adverse reactions. Following radiotherapy, the patient’s right limb muscle strength improved from grade IV to grade V−.

**Figure 2:**
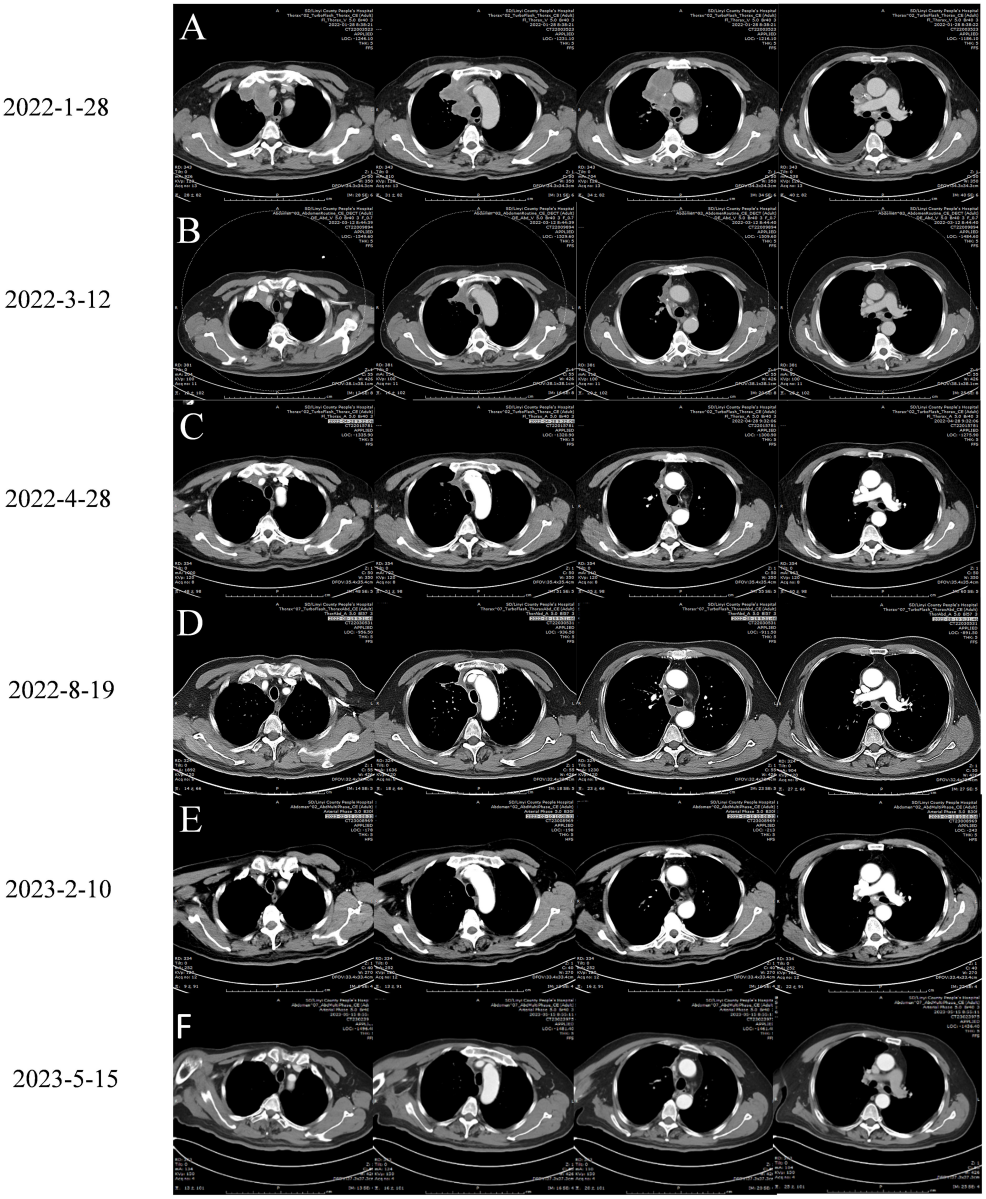
Chest enhanced CT images of the patient before and after treatment with Tislelizumab Combined with Anlotinib:arterial phase imaging. **(A)** January 28, 2022 is the chest CT image before treatment. **(B)** On March 12, 2022, chest CT images of two cycles of treatment with Tislelizumab Combined with Anlotinib. **(C)** On April 28, 2022, chest CT images were obtained after 4 cycles of treatment with Tislelizumab Combined with Anlotinib. **(D)** Chest CT images of 9 cycles of treatment with Tislelizumab Combined with Anlotinib on August 19, 2022. **(E)** On February 10, 2023, a chest CT image was obtained. The patient underwent a total of 13 cycles of treatment with trastuzumab and anlotinib, as well as 2 doses of trastuzumab monotherapy. **(F)** Chest enhanced CT scan on May 10, 2023, patient has terminated treatment for 3 months.

**Figure 3:**
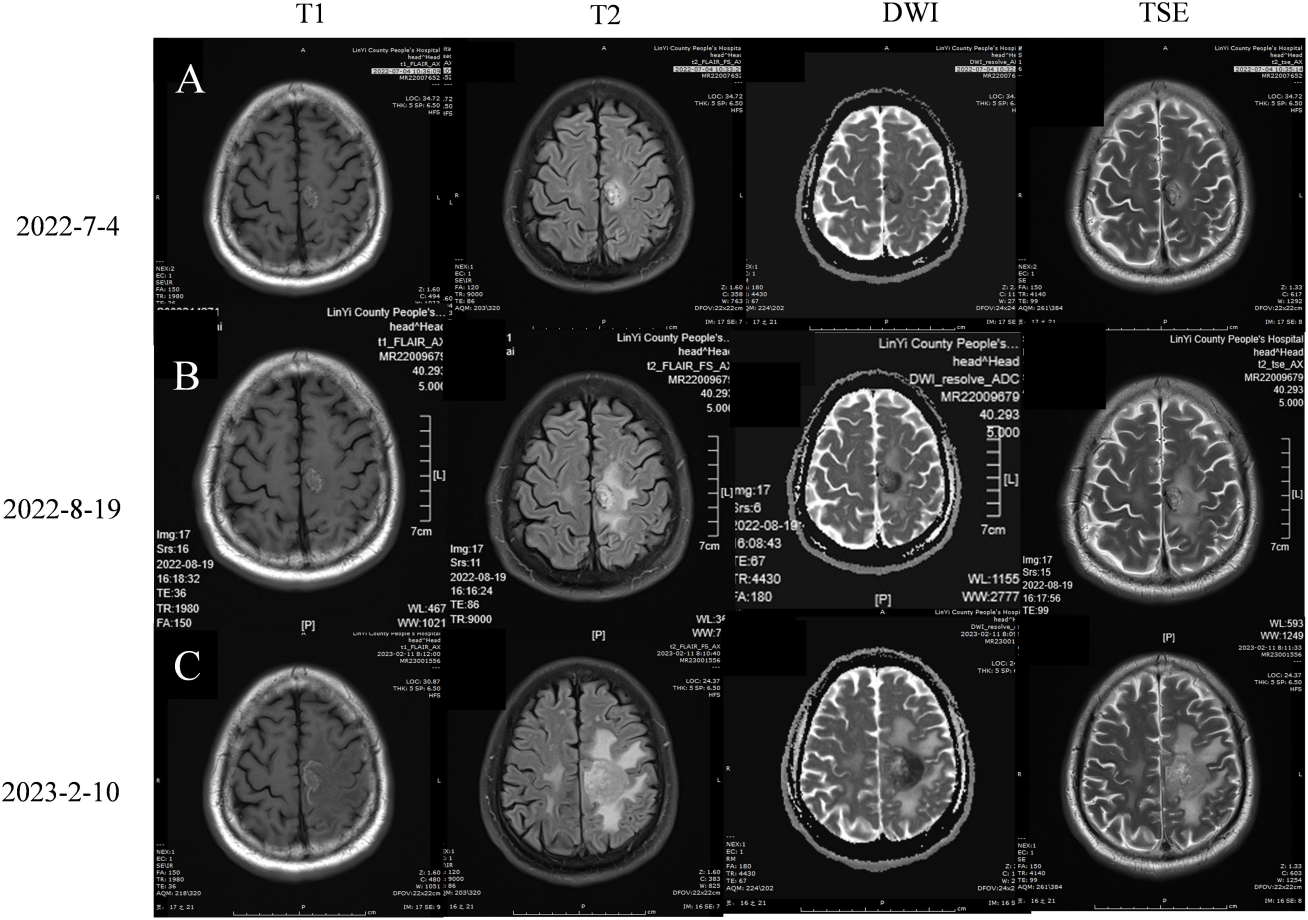
Brain magnetic resonance imaging **(A)** 2022-7-4 Brain MRI plain scan + enhancement +DWI:On the left parietal lobe, there is a circular lesion with short T1, long T2, and high FLAIR abnormal signals, with clear edges. The DWI shows high and low signals, measuring 1.4 * 1.6cm in size. Localized cortical discontinuity and blurred boundary are observed in the left parietal bone. **(B)** 2022-8-19 Brain MRI plain scan + DWI+MRA: On the left parietal lobe, there is a circular lesion with short T1, long T2, and high FLAIR abnormal signals, with clear edges. DWI shows high and low signals, with no significant changes compared to the 2022-7-4 image. Localized cortical discontinuity and blurred boundary are observed in the left parietal bone, with no significant changes compared to the previous image. The basilar arteries were in normal shape and the blood flow signal was not abnormal. The wall of the M1 section of the bilateral middle cerebral artery is rough and straight, and the branches below the M2 section are reduced and thin. The blood flow signal was weakened in the anterior part of the left anterior cerebral artery traffic, and the blood flow signal was weakened in the bilateral posterior cerebral artery, which was thin and out of shape. Imaging diagnosis: 1. Multiple ischemic lesions in the brain; 2. The MR results are consistent with left frontal lobe brain metastasis and left parietal bone metastasis. It is consistent with MRA manifestation of atherosclerosis. **(C)** 2023-2-10 Brain MRI plain scan + enhancement +DWI: In the left frontal parietal lobe, short T1 and long T2 high FLAIR abnormal signal foci were found, such as circular ones, with clear edges and high and low DWI signals. The maximum cross section was about 3.3x3.4cm, which was larger than the range of 2022-8-19, and a long flake T2 edema band was seen around it. The lesion of the left parietal bone showed irregular and mild enhancement, which was larger than that of 2022-8-19.

**Figure 4:**
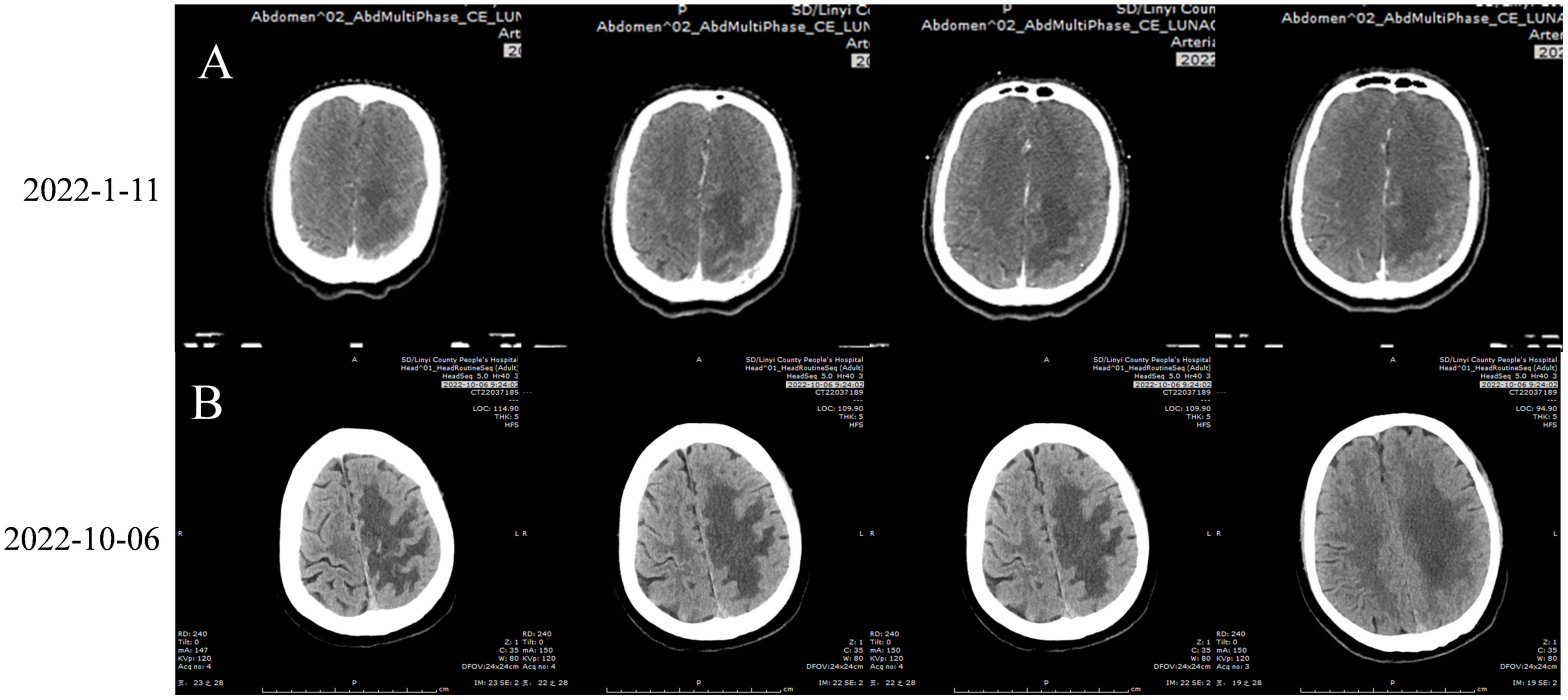
**(A)** 2022-1-11 Brain Enhanced CT: A circular lesion with a size of about 1.6×1.8cm was observed at the junction of gray matter in the left parietal lobe, with large sheet edema surrounding the lesion and a centered midline structure. Irregular bone destruction on the left parietal bone. **(B)** 2022-10-6 Brain Enhanced CT:A circular lesion with a size of approximately 1.4 × 1.6cm can be seen in the gray white border area of the left parietal lobe. The lesion is surrounded by large areas of edema, and the midline structure is centered. Irregular bone destruction area was observed in the left parietal bone, with no significant changes compared to before (January 11, 2022). **Figure of Timeline.** Timeline of treatment course of case reports. PR, partial reaction; PD, disease progression.

On January 28, 2022, a follow-up CT scan of the neck and chest revealed a mass-like soft tissue density lesion in the upper right mediastinum, with a maximum cross-sectional area of approximately 5.8 × 7.1 cm, clear boundaries, and an uneven density. Imaging diagnosis presented right upper mediastinal lung cancer (**Figure 2A**). Between January 29 and July 28, 2022, treatment with “tislelizumab 200 mg d0 + anlotinib 12 mg (days 1–14)” was administered for nine cycles, with no significant adverse reactions. The muscle strength of the patient’s right limb was maintained at grade V−. Enhanced chest CT scans on March 12 and April 28, 2022, showed a significant reduction in the mediastinal tumors, and the efficacy was evaluated as a partial response (PR) (**Figures 2B, C**). On July 4, 2022, enhanced brain MRI showed a left frontal lobe metastatic tumor measuring 1.4 × 1.6 cm (**Figure 3A**), which was reduced in volume compared with that of the brain CT image on January 11, 2022 (1.8 × 1.6 cm) (**Figure 4A**).

On August 19, 2022, the patient was admitted to the hospital due to a decrease in the muscle strength of the right limb, classified as grade IV. Cervical MRI, routine blood tests, liver and kidney function assessments, and blood coagulation studies revealed no abnormalities. The enhanced chest CT scan on August 19, 2022 (**Figure 2D**) showed a slightly reduced lesion in the upper right mediastinum compared with the image on April 28, 2022 (**Figure 2C**). The brain plain MRI scan, diffusion-weighted imaging (DWI), and magnetic resonance angiography (MRA) exhibited no significant changes in the left frontal parietal lobe metastasis (**Figure 3B**) and the left parietal bone metastasis compared with the image on July 4, 2022 (**Figure 3A**). However, increased edema was observed in the surrounding tissue of the tumor. The MRA diagnosis indicated 1) multiple ischemic lesions in the brain and 2) findings consistent with MRA manifestations of atherosclerosis. The patient consulted at Shandong Provincial Hospital, with the craniocerebral MRI performed on August 19, 2022. The consultation suggested 1) multiple ischemia and infarction in the brain and 2) MRI findings of left frontal lobe metastatic tumor consistent with shrinkage after radiotherapy and a small amount of blood infiltration. Considering the small amount of bleeding in the left frontal lobe metastatic tumor, the patient was instructed to stop taking anlotinib and to receive twice immunotherapy with tislelizumab 200 mg on August 19 and September 15, 2022.

The patient was hospitalized on October 6, 2022, due to a further decrease in the right limb muscle strength to V−. Routine blood tests, the liver and kidney function, and blood coagulation were all normal. The patient underwent a cranial CT scan, and a circular lesion measuring approximately 1.4 × 1.6 cm (**Figure 4B**) was observed in the gray white border area of the left parietal lobe, with little change compared to before (**Figure 4A**). Large areas of edema were detected around the tumor tissue. An irregular bone destruction area was observed in the left parietal bone, with no significant changes compared to before. The patient did not undergo a repeat cranial MRI due to financial constraints. Cranial CT revealed no significant cerebral hemorrhage or tumor enlargement; however, the cerebral edema had worsened. Combined with the decreased right muscle strength of the patient, the tumor activity might have increased. Therefore, on October 6 and 28, 2022, the patient was treated with tislelizumab 200 mg and anlotinib 12 mg, days 1–14, for two cycles. Following a 1.5-month interruption in treatment due to the coronavirus disease 2019 (COVID-19) outbreak, the patient resumed treatment with tislelizumab 200 mg and anlotinib 12 mg on days 1–14 for two cycles, administered on December 12, 2022, and January 11, 2023. During this period, the muscle strength of the patient’s right limb has remained stable at grade IV−.

The patient was admitted to the hospital on February 10, 2023, with a decrease in right muscle strength to grade III. The enhanced chest CT performed on February 10, 2023 (**Figure 2E**), showed a stable tumor size in the right upper mediastinum compared with the image on August 19, 2022 (**Figure 2D**). On February 10, 2023, the cranial MRI showed that the maximum cross-sectional area of the left frontal parietal lobe tumor was approximately 3.3 × 3.4 cm (**Figure 3C**), which was larger than the range on August 19, 2022 (**Figure 3B**). The edema of the surrounding tissues and the left parietal bone metastasis increased compared with those in the previous images. Due to financial constraints, the patient declined genetic testing for lung cancer, including *EGFR*, *ALK*, and *ROS1*, as well as surgery for the brain metastasis and further brain radiation therapy. Subsequently, the patient discontinued antitumor treatment following local progression in the brain.

**Figure 5:**
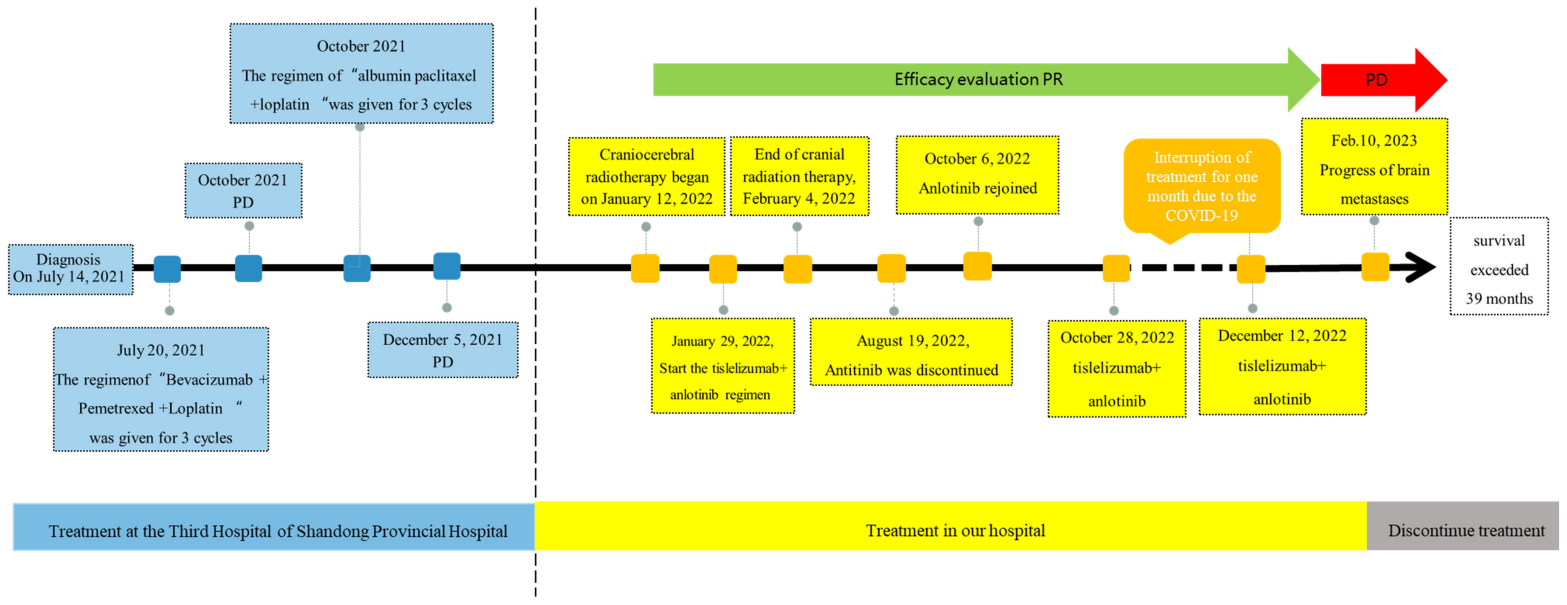
Figure of Timeline. Timeline of treatment course of case reports. PR, partial reaction; PD, disease progression.

On May 15, 2023, the patient’s chest CT examination showed a stable chest tumor (**Figure 2F**), and the patient did not return to the hospital for further examination or treatment since then. A telephone follow-up on October 10, 2024, suggested that the patient was still alive, but completely paralyzed in the right limb. Despite a decrease in the right muscle strength and a worsening brain edema after August 19, 2022, the size of the brain metastasis remained stable until February 10, 2023, when a significant increase was observed. Ultimately, it was hereby believed that the combination regimen of tislelizumab and anlotinib provided this patient with 12 months of progression-free survival (PFS), resulting in a total survival exceeding 39 months. The diagnosis and treatment process of this case is reflected in the form of a timeline (**Figure 5**).

## Discussion

Due to the rarity of PSC and the limited clinical data, its treatment is largely based on NSCLC protocols; however, many treatment schemes show limited efficacy (10). For early PSC, surgery remains the preferred treatment option (11, 12). Several studies have shown that neoadjuvant or adjuvant chemotherapy can provide significant survival benefits to postoperative patients with PSC (13, 14). However, there are also studies suggesting that perioperative chemotherapy is not beneficial for patients with stage I or stage IIa PSC. Therefore, perioperative chemotherapy for PSC is still controversial (14, 15). With the advancement of numerous molecular mechanism studies, PSC, such as other subtypes of NSCLC, has entered the era of immunotherapy and targeted therapy. Previous studies have shown that up to 72.3% of surgically resected PSC is PD-L1 positive (16) and that treatment with ICIs can prolong the PFS and overall survival (OS) in advanced PSC (17–19). Anti-vascular therapy can improve the tumor microenvironment and increase the infiltration of natural killer (NK) cells and antigen-presenting cells (APCs), thereby enhancing the efficacy of anti-programmed cell death protein 1 (PD-1) immunotherapy (20–23). Therefore, the combination of anti-angiogenic targeted therapy and ICIs might yield a synergistic effect and should be considered for patients with advanced PSC (24).

Tislelizumab is a humanized IgG4 anti-PD-1 monoclonal antibody that restores the clearance ability of T cells by blocking PD-L1/PD-L2-related cell signaling, leading to immune-related tumor cell death (25). Furthermore, the combination of tislelizumab and chemotherapy is now the standard first-line treatment for locally advanced and metastatic squamous NSCLC and mutation-negative non-squamous NSCLC (26). Anlotinib, a multi-target tyrosine kinase inhibitor (TKI), significantly improved survival in the third and subsequent lines of treatment for advanced NSCLC in China (27, 28). Studies have shown that anlotinib combined with ICIs can enhance survival in advanced NSCLC (29, 30). Therefore, it is important to investigate whether the integration of immunotherapy with anlotinib can offer survival benefits in PSC.

From January 29, 2022, to January 11, 2023, the patient received the treatment regimen of “tislelizumab 200 mg + anlotinib 12 mg on days 1–14 every 3 weeks.” During this period, anlotinib was stopped for two cycles due to bleeding in the brain metastasis, and all treatment was stopped for 1 month due to the COVID-19 outbreak. Over the course of more than a year, the patient’s chest tumor significantly reduced in size and almost disappeared, and the brain metastasis remained stable. Until February 10, 2023, the patient experienced local progression of the brain metastasis and terminated antitumor treatment due to economic reasons. Unexpectedly, a follow-up chest CT scan on May 15, 2023, showed that the chest condition of the patient remained stable, indicating the long-tailing effect of the protocol. The most recent telephone follow-up occurred on September 25, 2024, at which time the patient’s right limb was fully paralyzed, but with the patient still in good condition. Until now, the combination of tislelizumab and anlotinib has resulted in 12 months of PFS and a survival period of over 39 months. Undoubtedly, the patient experienced depression following the failure of two chemotherapy regimens. However, the patient was pleasantly surprised by the efficacy and survival benefits of the combination of immunotherapy with vascular targeted therapy.

This is acknowledged as the first case showing good results in a patient with brain metastasis of a PSC through the use of the tislelizumab and anlotinib regimen. While there are limited case reports on immunotherapy combined with anlotinib for PSC, the consistent results indicate that this combination can enhance disease control and prolong survival in patients. For example, Dai and his team treated a 75-year-old male patient diagnosed with PSC adrenal metastasis (cT4N3M1b, stage IVA) with sintilimab combined with anlotinib, with the patient achieving good results with tolerable adverse reactions (31). Jin et al. reported on a 62-year-old man with advanced PSC whose disease was controlled after 8 weeks of treatment with nivolumab and anlotinib (32). In the article by Wu et al., a 73-year-old male patient with locally advanced sarcomatoid lung cancer received pembrolizumab in combination with anlotinib for 15 cycles and experienced significant tumor reduction and OS exceeding 45 months (20). Wang also reported on a 29-year-old man diagnosed with lung sarcomatoid and adenocarcinoma with *EML4*–*ALK* gene fusion who experienced rapid tumor shrinkage after 6 weeks of treatment with alectinib combined with tislelizumab and anlotinib (21). In summary, the case and the report above showed that the combination of immunotherapy and anti-vascular therapy could provide survival benefits for patients with PSC. In addition, the patient’s cranial and cerebral metastases were controlled for approximately 1 year, indicating the efficacy of the combination of immunotherapy and vascular-targeted therapy in managing such metastases. However, further large-scale clinical trials and mechanistic studies are required to confirm these findings.

This study is also the first to report that sequential tislelizumab and anlotinib can achieve good therapeutic effects after cranial radiotherapy and that cranial radiotherapy can enhance the efficacy of immunotherapy and anti-angiogenic therapy. In 2023, Cai et al. reported on a female patient with advanced PSC who received treatment with tislelizumab and anlotinib combined with chest radiotherapy for over 2 years and achieved significant therapeutic effects (33). Previous studies have shown that the combination of radiotherapy and immunotherapy can strongly mobilize antitumor immunity through effector CD4^+^ and CD8^+^ T cells (34). It is extensively accepted that brain metastasis are more difficult to control than primary lung tumors, as it is difficult for drugs to pass through the blood–brain barrier. Therefore, it is also worth considering and researching whether cranial radiation therapy can enhance the blood–brain barrier permeability of drugs and improve the efficacy of immunotherapy by modulating the immune microenvironment.

The patient developed cerebral hemorrhage during treatment, which could not be directly related to anlotinib. While anlotinib increases the risk of bleeding in patients (6), instances of cerebral hemorrhage attributed to anlotinib are exceedingly rare (35). Moreover, a study involving 97 patients with lung cancer brain metastasis showed that anlotinib did not increase the risk of cerebral infarction or cerebral hemorrhage in patients with NSCLC compared with placebo (36). Similarly, there is no evidence that immunotherapy increases the risk of bleeding of anlotinib. Hemorrhage of brain metastasis is also associated with the abnormal growth of blood vessels inside the tumor, a weak blood vessel wall, or tumor necrosis (37). While it cannot be definitively concluded that the patient’s brain metastasis bleeding was caused by anlotinib, the monitoring and early detection of cerebral hemorrhage should be enhanced during treatment.

However, this case report has certain limitations. Firstly, the expression of PD-L1 and the status of gene mutations in patients are unknown. It is uncertain whether the effectiveness of this approach is associated with the high PD-L1 expression. Therefore, the therapeutic effects of tislelizumab and anlotinib in patients with different PD-L1 expression and gene mutation conditions are still unclear, necessitating large-scale cohort studies to verify the specific effects of tislelizumab and anlotinib in PSC. Secondly, due to the unanticipated favorable treatment response and the prolonged survival of the patient, a regimen of 3.0 Gy × 15 fractions of intensity-modulated radiotherapy for the brain was scheduled. If the patient initially received stereotactic radiotherapy for the brain or if the intensity-modulated radiotherapy dose was increased, the patient’s brain metastasis might have been more effectively controlled. Thirdly, sequential immunotherapy and vascular-targeted therapy were performed after cranial radiation therapy; however, the simultaneous implementation of these treatments might enhance the control effect of brain metastasis. This is also a question worth exploring. Finally, and most regretfully, the patient’s financial circumstances forced him to discontinue treatment, which might have otherwise improved his prognosis.

## Conclusion

Overall, the patient achieved good control in long-term PR and a survival period of over 39 months after receiving tislelizumab and anlotinib. While there is no evidence that anlotinib is associated with brain tumor bleeding in patients, vigilance for potential adverse events is still required. In addition, further exploration is needed for large-scale experiments and mechanistic studies on the combination of immunotherapy and anti-angiogenic therapy for PSC.

## Data Availability

The original contributions presented in the study are included in the article/**Supplementary Material**. Further inquiries can be directed to the corresponding author.

## Supplementary material

Supplementary Figure 1 Histopathological staining of the puncture biopsy specimen. **(A)** pathological diagnosis (lung biopsy): poorly differentiated carcinoma, combined with immunohistochemical results, conforms to pleomorphic carcinoma, some are poorly differentiated adenocarcinoma, and some are spindle cell carcinoma. Hematoxylin and eosin [H&E] staining, 100 ×. **(B)** CK7(+),100 ×. **(C)** Vimentin (mostly+),200 ×. **(D)** TTF-1 (lesion+), 100 ×, **(E)**. Napsin-A (lesion+), 200 ×, **(F)** ki-67 (40% -50%+), 100×. Figure B-F are all immunohistochemical staining.
