# Supplementary figures and images for: Dramatic response of advanced pulmonary sarcomatoid carcinoma to tislelizumab combined with anlotinib: a case report
Source: Front Oncol. 2025 Feb 7;15:1531700. doi: 10.3389/fonc.2025.1531700 (PMC11842246; doi:10.3389/fonc.2025.1531700)

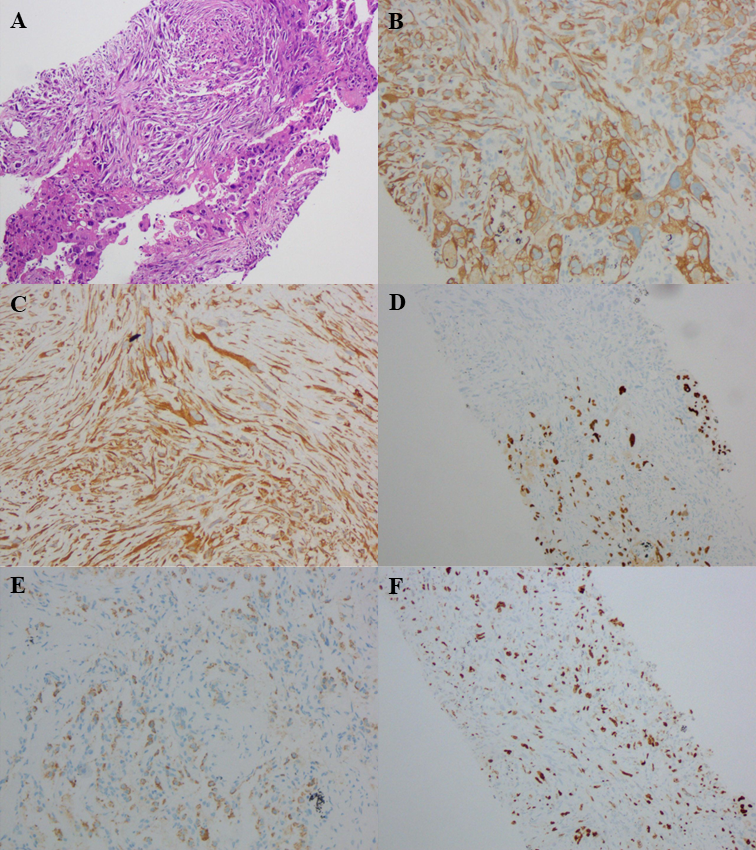

Supplement: Supplementary Figure 1 — Histopathological staining of the puncture biopsy specimen. (A) pathological diagnosis (lung biopsy): poorly differentiated carcinoma, combined with immunohistochemical results, conforms to pleomorphic carcinoma, some are poorly differentiated adenocarcinoma, and some are spindle cell carcinoma. Hematoxylin and eosin [H&E] staining, 100 ×. (B) CK7(+),100 ×. (C) Vimentin (mostly+),200 ×. (D) TTF-1 (lesion+), 100 ×, (E). Napsin-A (lesion+), 200 ×, (F) ki-67 (40% -50%+), 100×. Figure B-F are all immunohistochemical staining. [file Image1.tif]
